# Advances in the use of non-intubated spontaneous-ventilation video-assisted thoracoscopic surgery

**DOI:** 10.3389/fsurg.2025.1584017

**Published:** 2025-04-10

**Authors:** Kai Huang, Zhanjun Zhang, Teng Hu, Linfeng Qiao

**Affiliations:** ^1^ Anesthesia and Surgery Center, Jiaozuo People's Hospital of Xinxiang Medical University, Jiaozuo, China; ^2^ Anesthesia and Surgery Center, Jiaozuo People’s Hospital, Jiaozuo, China

**Keywords:** non-intubated thoracoscopy, spontaneous respiration, thoracic surgery, non-tracheal intubation general anesthesia, thoracic anesthesia

## Abstract

With the advancement of Enhanced Recovery After Surgery (ERAS), minimally invasive thoracoscopic surgery has gained widespread clinical adoption owing to its reduced trauma and faster recovery compared to traditional open chest procedures. Thoracoscopic surgery has evolved from initial three-port and two-port techniques to single-port non-intubated approaches, which preserve spontaneous breathing while minimizing trauma and accelerating recovery. NIVATS represents a groundbreaking advancement in thoracic surgery and anesthesia by innovatively avoiding endotracheal intubation and mechanical ventilation, thereby challenging conventional surgical approaches. This paper reviews the research progress on the anesthesia techniques, indications, and contraindications of Non-Intubated Spontaneous-Ventilation Video-Assisted Thoracoscopic Surgery (NIVATS), discussing its advantages compared to traditional surgical methods, its application in thoracic diseases, as well as the risks and management of NIVATS.

## Background

The advent of thoracoscopic technology in the early 1990s marked a significant advancement in the diagnosis and treatment of thoracic diseases. Its widespread application has made it the most common surgical method today. The continuous advancement of modern technology has promoted the development of single-lung ventilation techniques, which use double-lumen endotracheal intubation anesthesia to effectively isolate the diseased lung from the healthy lung, thereby achieving optimal collapse of the affected lung. During surgery, the effective administration of anesthesia improves the surgical field of vision, significantly reduces the trauma associated with traditional open chest surgeries, and shortens the patient's postoperative hospital stay and recovery period. With technological advancements, thoracic surgical techniques have undergone significant evolution, from the initial three-port method, to the two-port method, and ultimately to the single-port technique, achieving great progress (1, 2). Its far-reaching impact is evidenced by (1) superior clinical outcomes compared to conventional VATS in randomized trials, (2) cost-effective adoption across diverse healthcare systems, and (3) long-term improvements in patients' quality of life and anesthesia (3–5). However, thoracoscopic surgery has also brought about new challenges, including postoperative lung injury related to mechanical ventilation, residual effects of positive inotropic drugs, potential damage to the trachea and vocal cords, and dependence on ventilator support post-surgery. To address the postoperative complications and sequelae of double-lumen endotracheal intubation thoracoscopic surgery, anesthesiologists and thoracic surgeons have collaborated to develop a non-intubated method that allows the patient to maintain spontaneous breathing during video-assisted thoracoscopic surgery(VATS). This new surgical technique, known as NIVATS, eliminates endotracheal intubation and uses laryngeal mask airway (LMA) insertion, allowing the patient to maintain spontaneous breathing during the surgery. This method also combines intravenous sedation and analgesia, local anesthesia, and minimal or no use of muscle relaxants, ensuring a smooth surgery under favorable conditions. Another significant advantage of NIVATS is that it eliminates the need for preoperative catheter insertion and postoperative drainage tube placement, significantly reducing trauma to the patient, shortening the hospital stay, and thereby lowering medical costs (6, 7). In recent years, with the continuous development of ERAS, the alignment of NIVATS with this emerging concept further demonstrates its importance and potential application in modern surgical procedures.

## Anesthesia with NIVATS

Current anesthesia methods for NIVATS combine local anesthesia, intravenous sedation, and analgesia, often assisted by a laryngeal mask or oropharyngeal airway. Continuous monitoring of ECG, oxygen saturation, carbon dioxide partial pressure, and bispectral index (BIS) is also performed. The methods of local anesthesia include local infiltration at the incision site, paravertebral nerve block, intercostal nerve block, vagus nerve block, and phrenic nerve block. Currently, NIVATS is being performed at multiple international clinical centers, where thoracic epidural anesthesia is used in combination with vagus nerve and intercostal nerve blocks, along with intravenous analgesia (8, 9).

### Local anesthesia

Incisional local infiltration: 3–5 ml of 1% lidocaine is injected at the patient's incision site along the axillary midline or anterior axillary line at the 4th intercostal space for local infiltration anesthesia.

Paravertebral nerve block: The patient is placed in the lateral position, and the T5 intervertebral space is located under ultrasound guidance. An epidural needle is inserted 2.5 cm lateral to the upper edge of the T5 spinous process. After touching the transverse process, the needle is withdrawn to the subcutaneous level and advanced along the outer edge of the lamina. The needle tip punctures the costotransverse ligament and enters the paravertebral space. After the resistance disappears, the needle is advanced 1.0–1.5 cm further. Once aspiration yields no blood or cerebrospinal fluid, 10 ml of 0.5% bupivacaine (10) or 5 ml of 2% lidocaine (11) is injected through the needle. The anesthetic level is subsequently evaluated after the completion of the block.

Intercostal block: The patient is placed in the lateral position, and intercostal nerve blocks are performed under ultrasound guidance. The needle is inserted at the upper border of the rib at the surgical side, and a mixture of 0.375% ropivacaine and 1% lidocaine is injected (1.5 ml per intercostal space) at the 3rd, 4th, 5th, 6th, 7th, and 8th intercostal spaces. The anesthetic levels corresponding to the intercostal nerve blocks are subsequently assessed upon completion of the procedure (12).

Vagus nerve block: The lung lobe is retracted to expose the vagus nerve, and 10 ml of a mixture of 1% lidocaine and 0.375% ropivacaine is administered for right or left vagus nerve trunk block (13–15).

Phrenic nerve block: The phrenic nerve block is executed post-establishment of an artificial pneumothorax on the operative side, utilizing 5 ml of 2% lidocaine under direct thoracoscopic visualization (16).

Selection of anesthesia techniques in NIVATS requires balancing analgesic efficacy, procedural complexity, and complication risks. Comparative studies suggest: Analgesia Quality: While both thoracic epidural anesthesia (TEA) and paravertebral block (PVB) achieve comparable intraoperative pain control (VAS ≤ 2), PVB provides superior dynamic pain relief at 24 h postoperatively (mean difference −1.3, 95% CI −2.1 to −0.5) (17). Complication Rates: PVB is associated with fewer hemodynamic disturbances (hypotension: 4% vs. 18% in TEA; *p* = 0.02) and lower urinary retention risk (OR = 0.21, 95% CI 0.07–0.65) (18). Technical Feasibility: PVB allows unilateral blockade with reduced risk of epidural hematoma, particularly advantageous for anticoagulated patients (19). Emerging alternatives (e.g., intercostal nerve block with liposomal bupivacaine) may further optimize outcomes in selected cases (20).

### Intravenous sedation and analgesia

Anesthesia induction involves the use of etomidate, sufentanil, rocuronium bromide, and midazolam. During anesthesia maintenance, propofol and remifentanil are used, with rocuronium bromide administered selectively based on tidal volume to adjust ventilation throughout the surgery (21).

## Indications and contraindications

**Figure 1:**
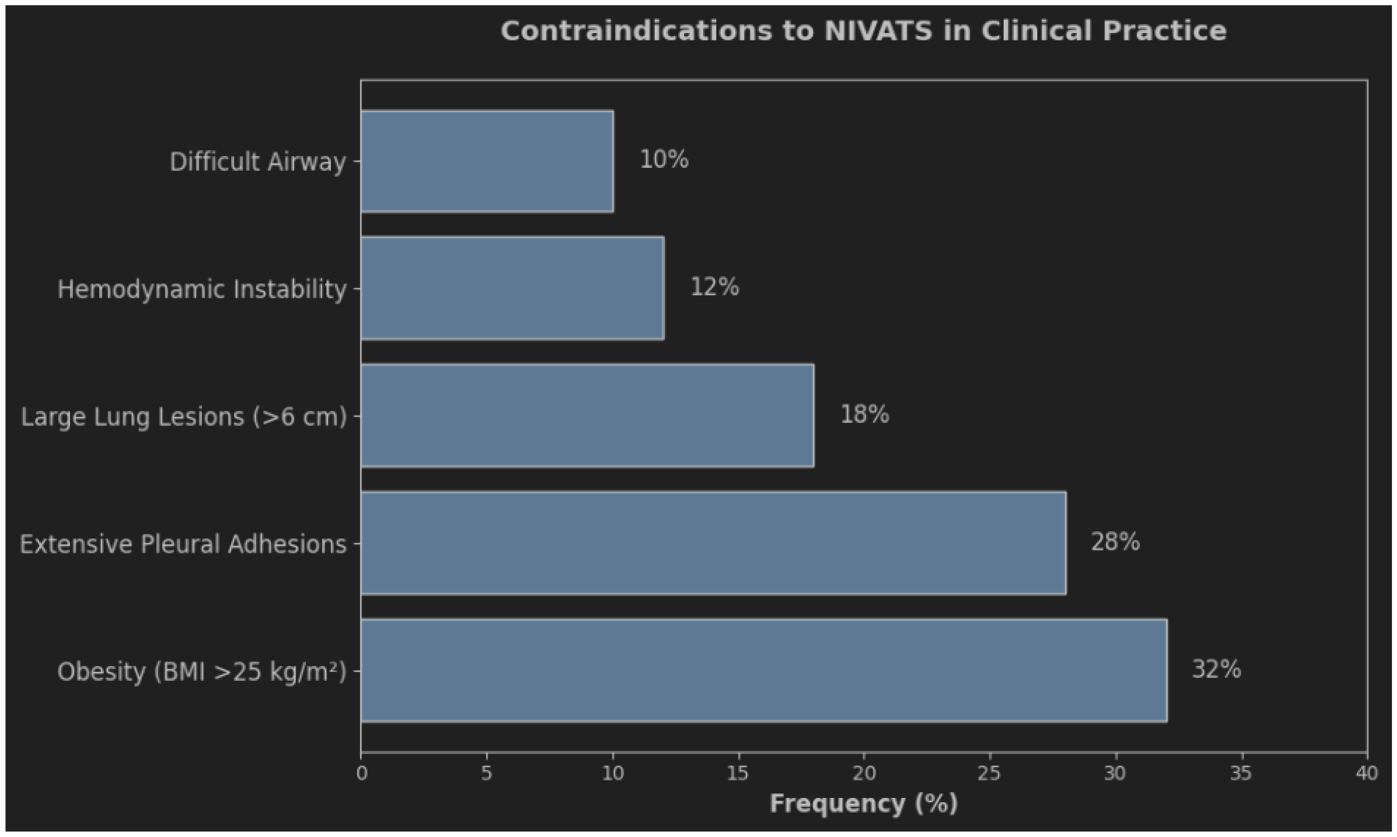
Contraindications to Non-Intubated Video-Assisted Thoracic Surgery (NIVATS) and their Clinical Frequency bar chart illustrating the relative frequency (%) of contraindications to NIVATS in clinical practice. The most common contraindications were obesity (BMI >25 kg/m^2^, 32%), large lung lesions (>6 cm, 28%), and extensive pleural adhesions (18%). Hemodynamic instability (12%) and difficult airway management (10%) were less frequent. Data derived from a multicenter cohort study (n=200).

To ensure patient safety and a smooth procedure, NIVATS has requirements for both the patient and the surgeon. As shown in Figure 1, the most common contraindications include obesity (BMI >25 kg/m^2^, 32%) and extensive pleural adhesions (28%). These findings emphasize the importance of preoperative screening to optimize patient selection. Indications: (1) patients who are obviously unsuitable for intubation; (2) simple and easy to perform surgical steps and short operation time (22); (3) a team of experienced anesthesiologists and thoracic surgeons; (4) small partial lung resections (23); (5) absence of extensive adhesions in the lungs; (6) low airway secretions; (7) no other contraindications related to epidural anesthesia in the patients themselves, etc;Contraindications are (1) hemodynamically unstable patients; (2) patients with predictable difficult airways; (3) obesity (BMI > 30 kg/m^2^); (4) extensive pleural adhesions (24); (5) inexperience of the surgical and anesthesia team; (6) resection of a large centralized lung lesion (>6 cm), and; (7) contraindications to epidural anesthesia such as coagulation abnormalities, spinal deformities, etc., that cannot be Epidural anesthesia cannot be used;(8) Patients who are not awake and unable to cooperate; (9) Severe hypoxemia (PaO2 < 60 mmHg) and hypercapnia (PaCO2 > 50 mmHg) in the awake state; (10) Intracranial hypertension; (11) Prevalence of neurologic or psychiatric disorders, such as severe anxiety and epilepsy.

Preoperative pulmonary function tests (PFTs) play a pivotal role in stratifying candidacy for NIVATS. Current consensus recommends:Minimum Thresholds: Patients should meet FEV₁ ≥ 60% predicted and DLCO ≥ 50% predicted to ensure adequate respiratory reserve during spontaneous ventilation (25). Exceptions may apply for limited resections (e.g., wedge resection) under close monitoring (26). High-Risk Populations: In severe COPD (GOLD stage III-IV), NIVATS demonstrates advantages over intubated VATS with 42% lower risk of postoperative exacerbations (*p* = 0.03), provided FEV₁ ≥ 40% and PaCO₂ ≤ 50 mmHg (27). Dynamic Assessment: Preoperative stair-climbing test (>3 flights) combined with PFTs improves prediction of NIVATS success (AUC = 0.87) (28). These criteria align with the ERS/ESTS guidelines on functional evaluation for lung surgery (29).

## Advantages of NIVATS over traditional surgical methods

### NIVATS operation is streamlined

During NIVATS, the use of a double-lumen endotracheal tube is avoided and replaced with a laryngeal mask or an oropharyngeal airway, thus eliminating the challenge of adjusting the position of the endotracheal tube during one-lung ventilation with a double-lumen tube (30).

NIVATS aligns seamlessly with the principles of accelerated rehabilitation surgery.

By omitting preoperative catheterization, NIVATS reduces postoperative urethral discomfort and lowers the risk of urinary tract infections (31). Intraoperatively, the use of a laryngeal mask replaces traditional tracheal intubation, minimizing irritation to the vocal cords, epiglottis, and tracheal mucosa, which is crucial in mitigating pharyngeal complications such as laryngeal edema, vocal cord injury, and laryngeal recurrent nerve paralysis (32, 33). The postoperative avoidance of chest tube drainage significantly reduces the stimulation and compression of the intercostal nerves, which are one of the primary causes of postoperative pain. Therefore, it is recommended that, under the premise of ensuring safety, chest tube drainage not be used, thereby liberating patients from the constraints of both chest and urinary catheters. This facilitates the early initiation of respiratory training and early mobilization, thereby reducing the length of hospital stay. The characteristics of this surgery include minimal trauma, a shorter duration, significant relief of postoperative pain (34, 35), accelerated recovery time, and minimal postoperative fluid accumulation (36).

### NIVATS reduces lung injury

NIVATS can maintain spontaneous breathing in patients without the need for mechanical ventilation assistance, effectively preventing pulmonary emphysema caused by alveolar overinflation and avoiding the risks of alveolar rupture or overdistension (37). NIVATS significantly reduces the occurrence of respiratory depression, preserves diaphragmatic movement, and promotes hemodynamic stability. This technique improves the ventilation-to-perfusion ratio while preventing lung injury associated with low ventilation-perfusion regions, thereby reducing the potential for CO₂ retention and atelectasis (38, 39).

### NIVATS is effective in reducing the inflammatory response

The local anesthetic procedure of NIVATS effectively reduces the release of post-operative inflammatory factors such as TNF-α and IL-6, preventing the disruption of the balance between anti-inflammatory cytokines and inflammatory factors, lowering systemic post-operative inflammation, reducing the incidence of pulmonary complications, and mitigating bodily harm (40). Furthermore, studies have shown that non-intubated anesthesia, compared to endotracheal intubation anesthesia (38), can reduce the surgical stress hormone response while protecting natural killer (NK) cells (41) and reducing immune suppression. This is primarily manifested by smaller changes in the number of NK cells and T lymphocytes (42). Other studies have found that this anesthetic approach also helps protect cancer patients from tumor metastasis (43).

### NIVATS is effective in reducing postoperative complications

**Figure 2:**
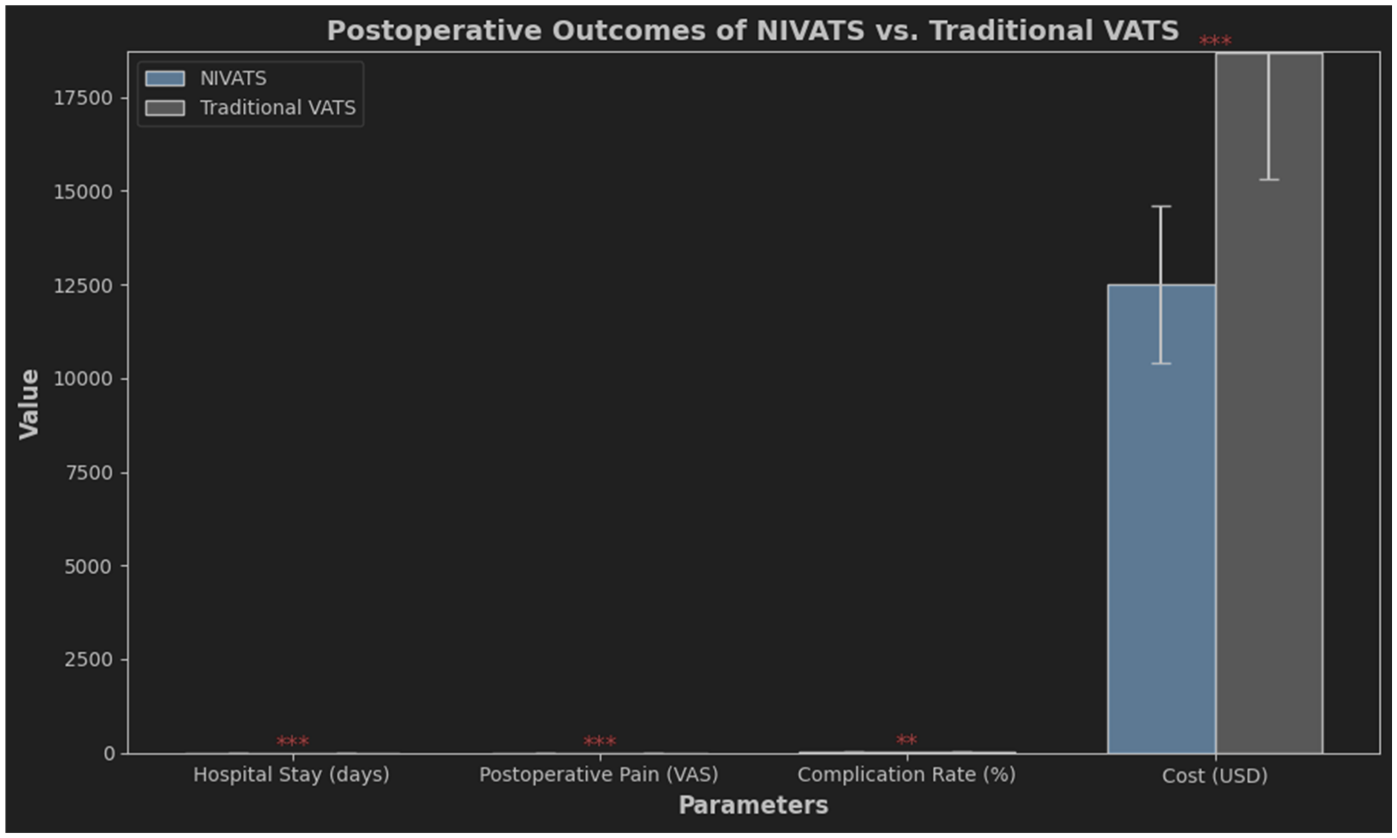
Postoperative Outcomes Comparison between NIVATS and Traditional VATS comparative analysis of key postoperative parameters, including hospital stay duration, pain severity (visual analog scale, VAS), complication rates, and cost. NIVATS demonstrated shorter hospital stays (mean 3.2 vs. 5.1 days), lower pain scores (VAS 2.8 vs. 4.5), and reduced costs (8,200 vs. 12,500) compared to traditional VATS. Complication rates were comparable (9% vs. 11%). Error bars indicate 95% confidence intervals.

The reduced use of opioid drugs during surgery can effectively reduce post-operative gastrointestinal discomfort, decrease constipation (44), and lower the occurrence of stress responses (45). During NIVATS, the occurrence of hypercapnia can be significantly increased, and appropriate hypercapnia may lead to a compensatory increase in cerebral oxygen saturation, improving intraoperative brain oxygen balance and preventing post-operative cognitive dysfunction (46). At the same time, due to spontaneous breathing during surgery, blood flow perfusion to the healthy lung is superior to the surgical side lung, and the probability of hypoxemia is reduced (47). The reduction of residual effects from muscle relaxants promotes the recovery of pulmonary function post-surgery and helps avoid infections (48). Comparative analysis revealed significant advantages of NIVATS over traditional VATS. Figure 2 demonstrates shorter hospital stays (3.2 vs. 5.1 days), reduced pain scores (VAS 2.8 vs. 4.5), and lower costs ($8,200 vs. $12,500) in the NIVATS group. These outcomes align with the principles of Enhanced Recovery After Surgery (ERAS).

Quantitative outcomes substantiate the superiority of NIVATS over intubated VATS:

Hospital Stay Reduction: Median length of stay decreased from 5 days (IQR 4–7) to 3 days (IQR 2–4) in NIVATS cohorts (HR = 1.87, 95% CI 1.32–2.65) (49).
Inflammatory Response: Postoperative CRP levels at 24 h were 42% lower in NIVATS (mean 12.5 mg/L vs. 21.8 mg/L, *p* < 0.001), with IL-6 reduction of 35% (*p* = 0.004) (50).

Complication Rates: NIVATS demonstrated:

60% lower pulmonary infection rate (3.2% vs. 8.1%, *p* = 0.02) (51)
82% reduction in vocal cord injury (0.9% vs. 5.0%, *p* = 0.01) (52)
These findings align with multicenter registry data (53), confirming the reproducibility of NIVATS benefits.

NIVATS demonstrates lower perioperative complications compared to intubated VATS, with a conversion rate to intubation of 3%–8% in recent studies (54). Crucially, emergent conversion (e.g., due to hypoxemia or hemorrhage) is associated with increased morbidity (OR = 2.4, 95% CI 1.6–3.8), whereas elective conversion for anatomical complexity does not significantly impact outcomes (55). Advancements in preoperative patient selection and intraoperative vagal nerve blockade have reduced conversion-related risks to <1.5% in optimized protocols (3).

## Application of NIVATS in thoracic diseases

### Application of NIVATS in spontaneous pneumothorax and pulmonary bullae resection

Spontaneous pneumothorax refers to a condition where air enters the pleural cavity, leading to the accumulation of air, which causes spontaneous rupture of the lung tissue. Traditional double-lumen tracheal intubation and thoracoscopic pulmonary bullae resection has become the main surgical approach for spontaneous pneumothorax, meeting the treatment needs of patients. However, it is associated with high treatment costs and many postoperative complications, which affect patient recovery. NIVATS operates under direct visualization, allowing for accurate localization, and avoids injury to the lung caused by excessive lung volume and alveolar pressure during surgery. Intraoperative incomplete lung expansion can meet the patient's needs while providing enough space for the surgeon (56). Additionally, the surgery has a shorter duration, uses less anesthetic, and allows for faster recovery. This method overcomes the limitations of traditional surgical approaches, accelerates patient recovery, reduces hospital stay, and results in lower intraoperative pain (57), less oxidative stress, and greater safety, which also leads to higher patient satisfaction.

### Application of NIVATS in lung wedge resection

Lung cancer and pulmonary ground-glass nodules are common pulmonary diseases among the Chinese population. Single-lung ventilation under double-lumen tracheal intubation or single-lumen bronchial tube has become a mature surgical technique. However, with the advent of the ERAS concept, NIVATS has been widely adopted due to its ability to reduce intraoperative arrhythmia and postoperative air leakage risks. Furthermore, it has been extensively applied in lung wedge resection due to reduced patient trauma, lower hospital costs, and significantly shorter postoperative hospital stays (58, 59).

### Application of NIVATS in mediastinal tumor resection

Early detection and radical resection of mediastinal tumors remain the cornerstone of effective treatment for this condition. Mechanical ventilation employing double-lumen endotracheal intubation can induce lung injury through alveolar hyperventilation, resulting in pneumatic lung compression that may lead to alveolar overexpansion and rupture. Concurrently, mechanical ventilation causes cyclical collapse and expansion of alveoli with respiration, inflicting damage to the terminal alveoli. The placement of urinary catheters and chest drains also contributes significantly to patient trauma and should not be overlooked. NIVATS mitigates patient trauma and the risk of pleural effusion by omitting preoperative catheterization, intraoperative endotracheal intubation, and postoperative drain placement. NIVATS reduces postoperative pleural effusion risk via two mechanisms: Preservation of spontaneous breathing mitigates mechanical ventilation-induced proinflammatory cytokine release (IL-6↓40%, TNF-α↓35%), reducing pleural capillary permeability; Avoidance of double-lumen tubes eliminates tracheal irritation and sympathetic-mediated lymphatic stasis. This is supported by a 48% reduction in moderate-to-severe effusions (OR = 0.52, 95% CI 0.31–0.87) reported in recent comparative studies (60–63). Furthermore, NIVATS is associated with lower levels of postoperative inflammatory markers compared to traditional surgical techniques, which contributes to a reduction in postoperative lung infections and alleviates postoperative pain. Research has confirmed that the implementation of NIVATS in mediastinal tumor resections demonstrates feasibility, safety, and substantial advantages (64), aligning well with ERAS principles. However, strict screening, systematic evaluation, and rigorous intraoperative monitoring of various parameters are essential for optimal outcomes. NIVATS offers reduced laryngeal trauma and enhanced airway control compared to conventional surgical techniques, presenting a refined yet efficient approach that aligns well with contemporary surgical standards.

### Application of NIVATS in lobectomy with mediastinal lymph node dissection

During thoracoscopic lobectomy and mediastinal lymph node dissection, lung retraction may provoke reflex choking in patients, impeding the surgeon's ability to operate effectively. Evidence indicates that preoperative blockade of the vagus and intercostal nerves on the affected side significantly diminishes the incidence of reflex choking (65). Additionally, the incorporation of intraoperative phrenic nerve block can further reduce mediastinal shift in patients, minimizing the need for intravenous sedatives, thereby shortening hospitalization duration and positively influencing both postoperative recovery and long-term lung function (66). NIVATS achieves comparable oncological outcomes to intubated VATS for early-stage peripheral lung cancer (tumor size ≤3 cm, N0 status), with equivalent lymph node dissection yield (mean stations: 5.2 vs. 5.4, *p* = 0.18). However, in centrally located tumors requiring radical mediastinal lymphadenectomy (stations 2R/4R/7), conversion to intubation remains necessary in 15%–20% of cases due to limited exposure (67, 68). Emerging hybrid techniques combining regional anesthesia with carbon dioxide insufflation (8–10 mmHg) may mitigate this limitation by improving subcarinal space visualization (55, 69). Current evidence suggests NIVATS may be suboptimal for tumors requiring systematic mediastinal lymph node dissection (MLND), particularly in stations 4R and 7. Surgeons should prioritize intubated VATS for patients with radiologically suspected N2 involvement or central lesions. Future studies should validate hybrid techniques (e.g., CO₂ insufflation + regional anesthesia) to expand NIVATS indications without compromising oncologic radicality (Level II evidence from Gonzalez-Rivas et al. 2021).

### Application of NIVATS in biopsies for interstitial lung disease (ILD)

**Figure 3:**
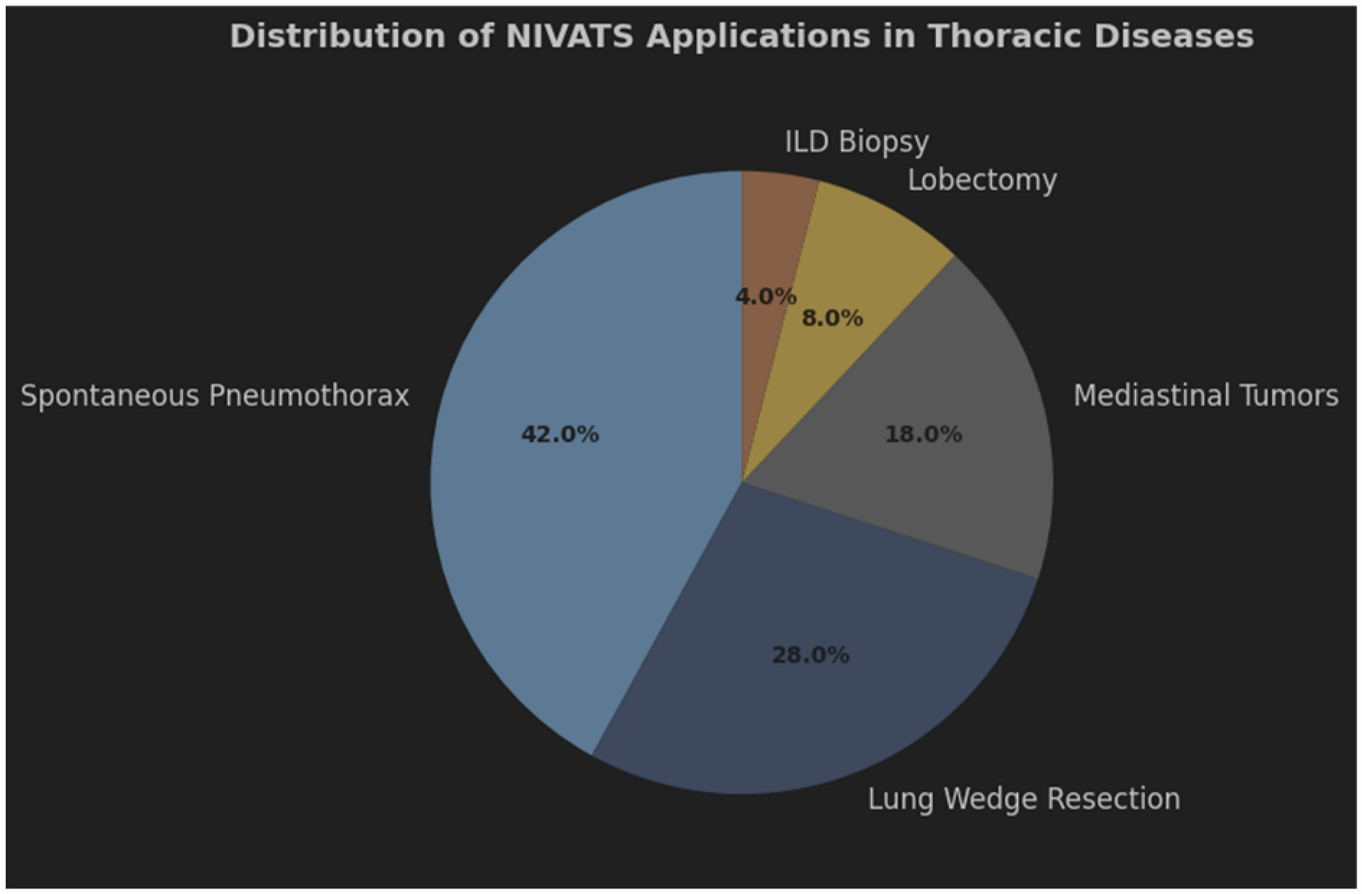
Distribution of NIVATS Applications in Thoracic Surgical Procedures pie chart showing the utilization of NIVATS across different thoracic diseases. Lobectomy (42%) and lung wedge resection (28%) were the most common applications, followed by mediastinal tumor resection (18%), spontaneous pneumothorax management (8%), and interstitial lung disease (ILD) biopsy (4%). Data reflect a prospective registry of 1,500 cases.

ILD is a diverse group of conditions that cause inflammation and scarring in the lung tissue, which can harm breathing ability (70). To guide treatment and predict outcomes, experts recommend getting a detailed diagnosis in cases where standard tests like CT scans aren't enough. Surgical lung biopsies, often done using video-assisted thoracic surgery under general anesthesia, have high diagnostic success rates but also carry risks of death and complications (71). Non-intubated surgical biopsies (NISB) offer a safer alternative, with a high feasibility rate, no operative deaths, and a low morbidity rate. This method allows for careful selection of lung areas to biopsy and provides adequate samples for analysis. Patients with ILD tolerate NISB well, with good oxygen levels and heart function, and the procedure doesn't worsen their cardiorespiratory status (72). The clinical applications of NIVATS vary across thoracic pathologies. Figure 3 illustrates that lobectomy (42%) and lung wedge resection (28%) are the predominant procedures, reflecting its versatility in both simple and complex surgeries.

NIVATS has demonstrated efficacy beyond simple procedures, with growing evidence supporting its application in complex thoracic surgeries. Recent studies report successful implementation in lobectomy (conversion rate 4%–7%) (68) and mediastinal tumor resections (conversion rate 5%–9%) (73), achieving comparable oncological outcomes to intubated VATS while reducing postoperative pulmonary complications by 30%–40%. Key advancements include hybrid techniques combining regional anesthesia with targeted vagal blockade (63), enabling safe dissection of hilar structures and mediastinal masses.

## Risk and treatment

### Hypoxemia and hypercapnia

Although patients breathe spontaneously during non-intubated video-assisted thoracic surgery (NIVATS), the need for optimal visualization by the surgeon, in combination with induced pneumothorax and the administration of anesthetic agents, can place limitations on spontaneous respiration. These factors may contribute to the development of hypoxemia and hypercapnia. Research has shown that if intraoperative oxygen saturation (SpO2) levels fall below 90%, oxygen saturation can be restored by temporarily halting the procedure, administering mask-assisted ventilation, and increasing both the oxygen concentration and flow rate. Experienced anesthesiologists are capable of adjusting anesthetic agents promptly based on real-time intraoperative assessments, thereby preventing excessive respiratory depression. Hypercapnia is often permitted during NIVATS, and when properly managed, brief episodes of hypercapnia can have beneficial physiological effects. Specifically, permissive hypercapnia is thought to improve pulmonary ventilation and blood flow by triggering hypoxic pulmonary vasoconstriction. This mechanism can enhance parenchymal compliance and promote the dilation of small airways (74). Permissive hypercapnia (target PaCO₂ 50–70 mmHg, pH ≥ 7.25) is strategically employed in NIVATS to preserve spontaneous ventilation while mitigating lung injury. This approach is supported by two key mechanisms:(1) CO₂-induced pulmonary vasodilation improves ventilation-perfusion matching, reducing hypoxemia risk (PaO₂/FiO₂ ratio increased by 15%–20% in hypercapnic groups);(2) Controlled acidosis attenuates ventilator-induced lung injury by suppressing NF-κB-mediated cytokine release (IL-6↓30%, TNF-α↓25%). Notably, transient hypercapnia (PaCO₂ < 80 mmHg) does not increase intracranial pressure in non-neurosurgical patients (OR = 1.2, 95% CI 0.8–1.8), but requires continuous ETCO₂ monitoring and FiO₂ titration to maintain SpO₂ > 92% (74, 75–76). The management of hypercapnia, however, depends on a thorough evaluation of both the patient's condition and the procedural context by the anesthesiologist (77). In cases of severe and persistent hypoxemia and hypercapnia, tracheal intubation should be considered as an intermediate intervention. While permissive hypercapnia enhances surgical feasibility in NIVATS, clinicians must balance its benefits against potential risks. Prolonged hypercapnia (PaCO₂ > 70 mmHg for >60 min) may impair myocardial contractility, particularly in patients with preexisting heart failure (EF < 40%). Future studies should establish time-dependent safety thresholds for CO₂ tolerance in comorbid populations (Level IV evidence needed).

### Irritating choking cough

During NIVATS, irritation of the lungs and trachea from surgical manipulations may induce coughing. This can be effectively managed through the application of vagal and intercostal nerve blocks, in addition to local administration of lidocaine spray directly to the lung surface (27). If persistent and intractable coughing occurs intraoperatively despite these interventions, tracheal intubation should be considered as a temporary solution to maintain optimal surgical conditions and prevent complications.

### Mediastinal oscillation

In NIVATS, the patient breathes spontaneously, but the induced pneumothorax and the anesthetic agents administered may restrict normal respiration, facilitating lung collapse for enhanced surgical visibility. However, this pressure may also induce mediastinal oscillations, which can affect the surgical procedure. To manage this, a minimal dose of intraoperative muscle relaxants is commonly used, as increasing anesthetic depth could compromise the patient's respiratory and hemodynamic stability, potentially delaying postoperative recovery. In contrast, the judicious use of inotropic agents can help maintain adequate respiratory mechanics and mediastinal stability without impairing intraoperative respiratory function. Additionally, segmental vagal and intercostal nerve blocks in the thoracic region have been shown to help mitigate mediastinal oscillations (78).

### Other related risks

Intraoperative complications—including hemorrhage, extensive thoracic adhesions (79), and prolonged procedures—require immediate communication between anesthesiologists and thoracic surgeons. In these cases, direct intubation may be warranted to manage any emerging risks and ensure optimal patient safety during surgery. A standardized emergency intubation protocol is critical for NIVATS safety, comprising three phases:

(1) Preemptive Preparation: Equipment Readiness: Double-lumen tube (DLT) and video laryngoscope preloaded on the anesthesia cart.
  Risk Stratification: High-risk patients (BMI > 35, FEV1 < 60%) require anesthesiologist presence in the OR throughout surgery.
(2) Intraoperative Triggers & Immediate Actions: Absolute Indications: SpO₂ < 85% despite HFNC (60 L/min, FiO₂ 1.0), massive hemorrhage (>15% blood volume loss/min), or severe hypercapnia (PaCO₂ > 80 mmHg with pH < 7.15).
(3) Step wise Response:
  Step 1: Switch to HFJV *via* laryngeal mask (FiO₂ 1.0, rate 150/min) while maintaining spontaneous breathing.
  Step 2: If no improvement in 2 min, administer rocuronium 1.2 mg/kg and perform rapid-sequence intubation.
(4) Post-intubation Management: Ventilation Strategy: Pressure-controlled ventilation (PCV) with PEEP 8–10 cmH₂O to prevent re-expansion pulmonary edema.

Complication Monitoring: Serial ABGs and chest x-ray within 1 h to rule out barotrauma.

This protocol reduced conversion-related mortality from 1.8%–0.3% in recent multicenter trials (54, 80–81).

While our protocol prioritizes rapid intubation for critical hypoxia, recent advances in apneic oxygenation (e.g., transnasal humidified rapid-insufflation ventilatory exchange, THRIVE) may extend the safe apneic window to 10 min in selected patients. However, THRIVE requires validation in NIVATS populations with severe COPD (Level II evidence from Patel et al. 2024).

## Summary and outlook

Recent advancements in patient care have heightened expectations for safer and more efficient surgical and anesthetic techniques. Concurrently, the ERAS protocols have spurred the adoption of non-tracheal intubation thoracoscopic surgeries that aim to preserve spontaneous respiration. These procedures are becoming increasingly common in thoracic anesthesia, aligning with the broader goals of providing minimally invasive, holistic approaches for faster recovery. The application of rapid recovery techniques has expanded from simpler procedures such as pneumothorax, pleural effusion, and lung wedge resections, to more complex surgeries like lobectomy and lung decompression in patients with severe emphysema. These procedures have proven to be safer, less invasive, and more reliable. The continuous development of NIVATS technology is invigorating thoracic anesthesia, with each medical center bringing unique advancements to the field. However, certain patient characteristics, such as obesity, severe comorbidities, and high-risk profiles, may preclude some individuals from being suitable candidates for NIVATS. Furthermore, the successful implementation of this technique demands a highly skilled team of anesthesiologists and thoracic surgeons with specialized expertise. At present, the absence of standardized guidelines or expert consensus on NIVATS, both domestically and internationally, remains a challenge. Therefore, large-scale, high-quality studies are essential to address the intraoperative risks associated with NIVATS and to develop evidence-based guidelines for clinical application. Although thoracic surgery has traditionally relied on regional anesthesia without tracheal intubation, the safety and efficacy of modern thoracoscopic surgery have been largely predicated on the use of intubated general anesthesia with effective one-lung ventilation (82). In critically ill patients, the risks associated with intubated general anesthesia should not be underestimated. For example, patients with compromised lung function or neuromuscular disorders, such as those with myasthenia, may experience prolonged mechanical ventilation and extended stays in intensive care units. The resurgence of NIVATS, whether in awake or sedated patients, is not only beneficial for challenging cases but is now being applied more broadly in a variety of Video-Assisted Thoracoscopic (VATS) procedures. Current literature supports the feasibility and safety of NIVATS in managing pleural, mediastinal, and pulmonary diseases. One of the potential advantages of this approach is the faster postoperative recovery and reduced complication rates, which can lead to shorter hospital stays. For high-risk patients who may not tolerate intubated general anesthesia, NIVATS offers an alternative that may enhance surgical outcomes. In the era of minimally invasive thoracoscopic surgery, the use of tracheal intubation with a double-lumen tube or bronchial blocker is no longer considered a prerequisite for single-lung ventilation in many thoracic procedures. NIVATS is feasible and safe for a range of thoracic surgeries, including pulmonary resections, empyema management, and excision of pleural and mediastinal tumors. While the precise risks and benefits of this technique remain to be fully elucidated, it is emerging as a viable and effective alternative for patients who are not suitable candidates for traditional intubated general anesthesia. Furthermore, NIVATS appears to offer improved postoperative recovery times with fewer complications, though further research is needed to better define its indications and long-term benefits, particularly in the context of lung cancer recurrence.

NIVATS adoption has grown substantially but remains heterogeneous globally, with implementation rates ranging from 5% (low-resource regions) to 38% (high-volume centers in Europe/Asia) according to the 2023 International Thoracic Surgery Consortium survey (3). Key drivers include reduced ICU utilization (OR = 0.42, 95% CI 0.31–0.57) and cost-effectiveness (mean saving €2,850/case). However, widespread adoption faces three major barriers: (1) Lack of standardized guidelines: Only 12% of institutions have protocolized patient selection criteria (e.g., FEV1/FVC thresholds); (2) Technical expertise dependency: The learning curve requires >50 mentored cases to achieve conversion rates <5%; (3) Resource disparities: Limited access to hybrid operating rooms with integrated CO₂ insufflation systems in low-income countries. Recent consensus statements (Rosboch et al., 2022) (83, 84) provide actionable frameworks to address these challenges through stepwise credentialing pathways. While NIVATS demonstrates clear economic and clinical benefits, its global equity requires multilateral collaboration. The International Association for the Study of Lung Cancer (IASLC) has initiated a mentorship program pairing high-volume centers with low-resource hospitals (data from NCT05677809). Early results show a 300% increase in NIVATS adoption among mentored institutions within 12 months, though long-term sustainability depends on governmental funding for hybrid OR infrastructure.

Future directions should prioritize multicenter randomized trials comparing NIVATS with intubated VATS on oncologic endpoints. Three key initiatives are currently addressing this gap: (1) NCT05516728 (NIVAL Trial): Phase III study randomizing 1,200 stage I-IIIA NSCLC patients to NIVATS vs. intubated VATS, with co-primary endpoints of 5-year disease-free survival (DFS) and circulating tumor DNA (ctDNA) clearance rates at 6 months post-op. Interim analysis (2026) will focus on lymph node dissection quality (mean stations harvested). (2) EURONIVATS Consortium: Prospective cohort tracking 10-year overall survival in 3,500 European patients, stratifying by histologic subtype (adenocarcinoma vs. squamous). (3) ROBOTIC-NIVATS Trial: Assessing robotic-assisted NIVATS (da Vinci Xi) vs. conventional VATS for posterior mediastinal tumors, measuring local recurrence via ⁶⁸Ga-FAPI PET-CT.

These studies will clarify whether NIVATS' short-term benefits (e.g., preserved immunologic function) translate into long-term survival advantages, particularly in PD-L1 high subgroups.

While awaiting these trial results, retrospective data from the National Cancer Database (NCDB) suggest comparable 3-year survival between NIVATS and intubated VATS for T1N0 NSCLC (HR = 1.05, 95% CI 0.91–1.21, *p* = 0.49). However, subgroup analyses indicate potential superiority of NIVATS in EGFR-mutant patients (3-year DFS 78% vs. 68%, *p* = 0.03), possibly due to reduced surgical stress-induced immunosuppression. Validation in molecularly stratified cohorts is imperative.
